# Differences in clinical characteristics between adolescents and young adults with perinatally and sexually acquired HIV in the Asia‐Pacific region

**DOI:** 10.1002/jia2.26400

**Published:** 2025-04-08

**Authors:** Phatharajit Phatharodom, Alan Maleesatharn, Tavitiya Sudjaritruk, Suwimon Khusuwan, Kathy Petoumenos, Linda Aurpibul, Romanee Chaiwarith, Michelle L. Giles, Du Tuan Quy, Smita Nimkar, Alvina Widhani, Junko Tanuma, Matthew Law, Annette H. Sohn, Kulkanya Chokephaibulkit, IeDEA Asia‐Pacific

**Affiliations:** ^1^ Department of Preventive and Social Medicine Faculty of Medicine Siriraj Hospital Mahidol University Bangkok Thailand; ^2^ Siriraj Institute of Clinical Research and Department of Pediatrics Faculty of Medicine Siriraj Hospital Mahidol University Bangkok Thailand; ^3^ Department of Pediatrics, Faculty of Medicine Chiang Mai University Chiang Mai Thailand; ^4^ Clinical and Molecular Epidemiology of Emerging and Re‐emerging Infectious Diseases Research Cluster, Faculty of Medicine Chiang Mai University Chiang Mai Thailand; ^5^ Chiangrai Prachanukroh Hospital Chiang Rai Thailand; ^6^ The Kirby Institute UNSW Sydney Sydney New South Wales Australia; ^7^ Research Institute for Health Sciences Chiang Mai University Chiang Mai Thailand; ^8^ Division of Infectious Diseases and Tropical Medicine Department of Medicine, Faculty of Medicine and Research Institute for Health Sciences Chiang Mai University Chiang Mai Thailand; ^9^ Department of Infectious Diseases The Alfred Hospital and Monash University Melbourne Victoria Australia; ^10^ Infectious Diseases Department Children's Hospital 1 Ho Chi Minh City Vietnam; ^11^ BJ Government Medical College – Johns Hopkins University Clinical Research Site Pune Pune India; ^12^ Faculty of Medicine Universitas Indonesia ‐ Dr. Cipto Mangunkusumo General Hospital Jakarta Indonesia; ^13^ National Center for Global Health and Medicine Tokyo Japan; ^14^ TREAT Asia/amfAR, The Foundation for AIDS Research Bangkok Thailand

**Keywords:** Asia, adolescents, health‐related outcomes, perinatally acquired HIV, sexually acquired HIV, young adults

## Abstract

**Introduction:**

We assessed the long‐term HIV‐related health outcomes of young adults with perinatally acquired HIV (PHIV) compared with those who acquired HIV through sexual transmission in the Asia‐Pacific region.

**Methods:**

We conducted a cross‐sectional study using data from three paediatric and adult cohorts within the International epidemiology Databases to Evaluate AIDS (IeDEA) Asia‐Pacific consortium. This study included data from 12 countries, collected between 1991 and 2021. Young adults with available data who had been on antiretroviral therapy (ART) for at least 1 year were included. Analyses were conducted at ages 18 and 25 years and compared by route of HIV acquisition. Factors associated with viral suppression (<200 copies/ml) at age 25 were identified using logistic regression.

**Results:**

There were 1333 individuals included at age 18 (96% with PHIV: 46% male) and 305 at age 25 (27% with PHIV; 75% male). Compared to those with sexually acquired HIV at age 18, those with PHIV had a longer median duration of ART (10 vs. 4 years, *p<*0.001), higher current CD4 count (606 vs. 462 cells/mm^3^, *p =* 0.001), were shorter (height 158 vs. 166 cm, *p<*0.001), with more hypercholesterolemia (20% vs. 5%, *p* = 0.031) and hypertriglyceridemia (29% vs. 6% mg/dl, *p* = 0.003). At age 25, differences in duration of ART (15 vs. 3 years, *p<*0.001), male height (165 vs. 173 cm, *p =* 0.009) and proportion with hypertriglyceridemia (38% vs. 15%, *p =* 0.002) were observed. HIV viral suppression did not vary by mode of acquisition (89% vs. 87% at age 18; 91% vs. 85% at age 25). At age 25, living in Thailand (adjusted odds ratio [AOR] 6.05, 95% confidence interval [CI] 1.95−18.80) and use of integrase inhibitor‐based regimens (AOR 5.20, 95% CI 1.62−16.65) or protease inhibitor‐based regimens (AOR 2.62, 95% CI 1.01−6.79) were associated with viral suppression.

**Conclusions:**

Young adults with PHIV who survived to ages 18 and 25 were more likely to have stunted growth but had similar viral suppression to those with sexually acquired HIV in our regional cohorts. However, viral suppression rates remained lower for all relative to the UNAIDS goal of 95%, and measures to improve treatment outcomes are needed for young adults.

## 1 INTRODUCTION

In 2022, approximately 1.7 million adolescents aged 10–19 and 3.1 million young adults 15–24 were living with HIV [1]. Although antiretroviral therapy (ART) has improved the survival of those with perinatally acquired HIV (PHIV) [2], several studies have demonstrated poor treatment outcomes relative to those who acquired HIV later in life [3, 4, 5, 6, 7]. Complications have often been due to delayed treatment initiation in early childhood, adverse events from prolonged use of ART and eventual treatment fatigue in adolescence, resulting in high rates of virological failure and drug resistance. As these surviving adolescents move into young adulthood, it is increasingly important to better understand their risks for co‐morbidities, particularly in the era of improved access to safer and more efficacious ART.

In contrast, older adolescents and young adults who acquired HIV through risk behaviours have had shorter durations of living with HIV and ART exposure. However, they may live with different factors associated with HIV risk (e.g. substance use) and social challenges (e.g. lack of family support), which can lead to poor adherence and loss to follow‐up [8, 9, 10].

Identifying differences between young adults with perinatally or sexually acquired HIV can guide the development of optimal services and supporting systems. Using Asia‐Pacific HIV cohort data, we aimed to evaluate differences in health outcomes between these two groups at two key milestones: (1) at age 18, when adolescents with PHIV in the region would transition from paediatric to adult HIV care, and (2) at age 25, after the end of the period of young adulthood when they are expected to have established stable self‐care routines and achieved greater independence under adult care. Previous research in Thailand has shown high loss to follow‐up and mortality rates among individuals with PHIV during the transition to adulthood [11]. Studying youth across these age milestones will help to clarify these patterns and guide the development of tailored interventions and clinical management strategies to prevent adverse health outcomes during these critical transitions.

## 2 METHODS

### 2.1 Data sources

This multicentre, retrospective, cross‐sectional study includes data from people with HIV in the Asia‐Pacific region contributed through three databases of the International epidemiology Databases to Evaluate AIDS (IeDEA) Asia‐Pacific cohort consortium.

1. TREAT Asia Pediatric HIV Observational Database (TApHOD): children with HIV enrolled at ≤18 years and in care at 19 sites from six countries (Cambodia, India, Indonesia, Malaysia, Thailand, Vietnam).
2. TREAT Asia HIV Observational Database (TAHOD): adults with HIV in care at 21 sites in 12 countries (Cambodia, China [including Hong Kong SAR], India, Indonesia, Japan, Malaysia, Philippines, Singapore, South Korea, Taiwan, Vietnam, Thailand).
3. TREAT Asia HIV Observational Database‐Low Intensity Transfer (TAHOD‐LITE): adults with HIV at seven sites in seven countries (Cambodia, China [including Hong Kong SAR], India, Indonesia, South Korea, Vietnam, Thailand).

The principal investigator at each site was responsible for local data management. The Kirby Institute, UNSW, Sydney, Australia serves as the regional data centre, managing data and quality control activities. De‐identified individual clinical data were transferred electronically to the regional data centre through standardized formats and following confidentiality requirements. Institutional review board approvals were obtained at the sites, the regional data centre: the Kirby Institute and the coordinating centre at TREAT Asia/amfAR—The Foundation for AIDS Research. Informed consent was exempted because the study was a retrospective analysis of data collected in a programmatic setting.

### 2.2 Inclusion criteria

The inclusion criteria were as follows: (1) young adults with HIV who had available data at 18 and/or 25 years of age in cohort databases as of 30 April 2021; (2) documented route of HIV acquisition as perinatal or sexual (e.g. acquisitions through blood transfusion or intravenous drug use were excluded); and (3) exposure to ART for at least 1 year based on clinical records.

### 2.3 Study outcomes

The primary objective was to evaluate differences in clinical characteristics between individuals with PHIV and sexually acquired HIV, including demographic characteristics, health‐related factors and HIV‐related factors. The analysis was performed at two time points: age 18 and age 25. The secondary outcome was to identify factors associated with viral suppression at age 25, defined as an HIV viral load <200 copies/ml on two consecutive measurements, taken at least 3−12 months apart.

### 2.4 Statistical analysis

Univariable and multivariable logistic regression analyses were conducted to identify factors associated with viral suppression. Variables identified through a comprehensive literature review and expert opinion were selected *a priori*, including demographics (sex and country), type of HIV acquisition, ART regimens and immunologic response (CD4 count). These variables were then entered into multivariable logistic regression models using a forward selection approach with a significance threshold of 5% for selecting variables for inclusion in the final model. The missing values were excluded from the models as there was a minimal amount of missing data observed across the dataset. As the strategy of ART initiation for all regardless of CD4 levels rolled out in the region in 2015 and most with PHIV reached age 25 after 2015, the analysis at age 25 was performed using data since 2015. The significance level was set at *p* value less than 0.05. Data were analysed using STATA, version 17 (STATA Corp, College Station, TX, USA).

## 3 RESULTS

Out of a total of 32,491 individuals in the combined databases, there were 1333 and 305 who met the inclusion criteria at ages 18 and 25, respectively. Almost all of those at age 18 were retrieved from TApHOD (98%), and of those at age 25, 66% came from TAHOD and 24% from TAHOD‐LITE. At age 18, 96% had PHIV, 54% were female and 70% were from Thailand; 46% were receiving first‐line ART regimens, the median CD4 cell count was 599 cells/mm^3^ (IQR: 414, 788) and 88% were virally suppressed. Most other laboratory values were within normal limits (Table 1). In comparison to those with sexually acquired HIV, young adults with PHIV included a smaller proportion of males (45% vs. 71%, *p<*0.001), were shorter (154 vs. 161 cm for females, *p =* 0.009; 164 vs. 169 cm for males, *p<*0.001) and had a longer duration of ART (10 [IQR: 7, 13] vs. 4 [IQR: 2, 6] years, *p<*0.001), with a lower proportion receiving first‐line ART regimen (22% vs. 57%, *p<*0.001). Those with PHIV were more likely to have experienced WHO Clinical Stage 3 or 4 (59% vs. 31%, *p<*0.001). Although the CD4 cell counts were higher among young adults with PHIV (606 vs. 462 cells/mm^3^, *p =* 0.001), viral suppression rates were similar (89% vs. 87%, *p =* 0.774). While the majority of individuals in both groups used non‐nucleoside reverse transcriptase inhibitor (NNRTI)‐based regimens, protease inhibitor (PI)‐based regimens were more frequently used among those with PHIV, and integrase strand transfer inhibitor (INSTI)‐based regimens were more frequently used among those with sexually acquired HIV. Hypercholesterolemia and hypertriglyceridemia were more frequent among those with PHIV (hypercholesterolemia 20% vs. 5%, *p =* 0.031; hypertriglyceridemia 29% vs. 6%, *p =* 0.003).

**Table 1:** Characteristics and HIV‐related outcomes of adolescents and young adults with HIV at 18 years of age, by exposure status

Characteristics and HIV‐related outcomes	Total (*n* = 1333)	Perinatally acquired HIV (*n* = 1275)	Sexually acquired HIV (*n* = 58)	*p* value
**Sex**				
Female	722 (54.2%)	705 (55.3%)	17 (29.3%)	<0.001^*^
**Calendar year at age 18**				
Overall median (IQR)	2015 (2013, 2018)	2015 (2013, 2018)	2016 (2012, 2018)	0.199
**Country** ^a^				
Thailand	933 (70.0%)	889 (69.7%)	44 (75.9%)	0.294
India	113 (8.5%)	107 (8.4%)	6 (10.3%)	
Malaysia	104 (7.8%)	103 (8.1%)	1 (1.7%)	
Others	183 (13.7%)	176 (13.8%)	7 (12.1%)	
**Body mass index (kg/m^2^)**				
Available data	1259 (94.4%)	1208 (94.7%)	51 (87.9%)	
Underweight (< 18.5)	541 (43.0%)	519 (43.0%)	22 (43.1%)	0.968
Normal (18.5−24.9)	633 (50.3%)	607 (50.2%)	26 (51.0%)	
Overweight (> 24.9)	85 (6.7%)	82 (6.8%)	3 (5.9%)	
**Height (cm)**				
Available data	1161 (87.1%)	1121 (87.9%)	40 (69.0%)	<0.001^*^
Overall median (IQR)	158 (152, 164)	158 (152, 164)	166 (161, 172)	
Male; Median (IQR)	164 (159, 168)	164 (158, 168)	169 (165, 174)	<0.001^*^
Female; Median (IQR)	154 (150, 158)	154 (150, 158)	161 (153, 164)	0.009^*^
**Duration of ART (years)**				
Overall median (IQR)	9.8 (6.7, 13.0)	10.0 (7.0, 13.3)	3.7 (2.0, 6.0)	<0.001^*^
**Current ART regimens**				
NNRTI‐based	747 (56.0%)	704 (55.2%)	43 (74.1%)	<0.001^*^
PI‐based	469 (35.2%)	464 (36.4%)	5 (8.6%)	
INSTI‐based	92 (6.9%)	86 (6.7%)	6 (10.4%)	
Others	25 (1.9%)	21 (1.7%)	4 (6.9%)	
**Remaining in initial ART regimens**				
Yes	310 (23.3%)	277 (21.7%)	33 (56.9%)	<0.001^*^
**Current CD4 cell count**				
Overall median (IQR)	599 (414, 788)	606 (419, 792)	462 (282, 682)	0.001^*^
**Viral suppression (copies/ml)**				
Available data	1285 (96.4%)	1230 (96.5%)	55 (94.8%)	
Less than 200	1137 (88.5%)	1089 (88.5%)	48 (87.3%)	0.774
Less than 1000	1187 (92.4%)	1138 (92.5%)	49 (89.1%)	0.349
**History of worst WHO Staging**				
Available data	1205 (90.4%)	1179 (92.5%)	26 (44.8%)	
Stage 1	169 (14.0%)	154 (13.1%)	15 (57.7%)	<0.001^*^
Stage 2	332 (27.6%)	329 (27.9%)	3 (11.5%)	
Stage 3	403 (33.4%)	401 (34.0%)	2 (7.7%)	
Stage 4	301 (25.0%)	295 (25.0%)	6 (23.1%)	
**Total cholesterol (mg/dl)**				
Available data	832 (62.4%)	795 (62.4%)	37 (63.8%)	
Overall median (IQR)	166 (145, 192)	166 (146, 192)	155 (140, 170)	0.118
More than 200	158 (19.0%)	156 (19.6%)	2 (5.4%)	0.031^*^
**Triglyceride (mg/dl)**				
Available data	807 (60.5%)	772 (60.5%)	35 (60.3%)	
Median (IQR)	111 (80, 159)	112 (80, 162)	95 (71, 116)	0.170
More than 150	224 (27.8%)	222 (28.8%)	2 (5.7%)	0.003^*^
**High‐density lipoprotein (mg/dl)**				
Available data	407 (30.5%)	381 (29.9%)	26 (44.8%)	
Overall median (IQR)	46 (38, 57)	46 (38, 57)	47 (38, 54)	0.811
Low of HDL (female < 50, male < 40)	176 (44.0%)	170 (44.6%)	9 (34.6%)	0.320
**Low‐density lipoprotein (mg/dl)**				
Available data	262 (19.7%)	247 (19.4%)	15 (25.9%)	
Overall median (IQR)	96 (81, 113)	96 (80, 116)	94 (85, 100)	0.814
Low of LDL > 130	35 (13.4%)	35 (14.2%)	0 (0%)	0.117
**Fasting blood sugar (mg/dl)**				
Available data	771 (57.8%)	730 (57.3%)	41 (70.7%)	
Overall median (IQR)	84 (79, 90)	84 (79, 90)	87 (79, 91)	0.049^*^
FBS ≥ 100	58 (7.5%)	55 (7.5%)	3 (7.3%)	0.959
FBS ≥ 126	12 (1.6%)	11 (1.5%)	1 (2.4%)	0.639

Abbreviations: ART, antiretroviral therapy; FBS, fasting blood sugar; HDL, high‐density lipoprotein; HIV, human immunodeficiency virus; INSTI, integrase strand transfer inhibitor; IQR, interquartile range; kg/m^2^, kilogram per square metre; mg/dl, milligrams per decilitre; ml, millimetre; mmHg, millimetre of mercury; NNRTI, non‐nucleoside reverse transcriptase inhibitor; PI, protease inhibitor; WHO, World Health Organization.

^a^ Other countries include Cambodia, Hong Kong SAR, Indonesia, South Korea and Vietnam.

* *p* value <0.05.

At age 25, 27% had PHIV and 75% were male (Table 2). In comparison to those with sexually acquired HIV, those with PHIV had longer durations of ART (15 [IQR: 13, 17] vs. 3 [IQR: 2, 5] years, *p<*0.001), with a lower proportion receiving first‐line ART regimen (21% vs. 56%, *p<*0.001), and more experienced advanced WHO Stages (*p<*0.001). Height differences were observed among males (165 vs. 173 cm, *p =* 0.009), but not among females (155 vs. 150 cm, *p =* 0.190). CD4 cell counts and HIV viral suppression rates were similar, and patterns of ART regimens used were similar to those at age 18. A difference in the prevalence of hypertriglyceridemia was still observed (38% vs. 15%, *p =* 0.002).

**Table 2:** Characteristics and HIV‐related outcomes of adolescents and young adults with HIV at 25 years of age, by exposure status

Characteristics and HIV‐related outcomes	Total (*n* = 305)	Perinatally acquired HIV (*n* = 83)	Sexually acquired HIV (*n* = 222)	*p* value
**Sex**				
Female	76 (24.9%)	36 (43.4%)	40 (18.0%)	<0.001^*^
**Calendar year at age 25**				
Overall median (IQR)	2017 (2016, 2019)	2019 (2017, 2019)	2017 (2016, 2018)	<0.001^*^
**Country** ^a^				
Thailand	124 (40.7)	68 (81.9)	56 (25.2)	<0.001^*^
India	33 (10.8)	9 (10.9)	24 (10.8)	
Malaysia	15 (4.9)	3 (3.6)	12 (5.4)	
Others	133 (43.6)	3 (3.6)	130 (58.6)	
**Body mass index** (**kg/m^2^)**				
Available data	203 (66.6%)	81 (97.6%)	122 (55.0%)	
Underweight	31 (15.3%)	26 (32.1%)	5 (4.1%)	<0.001^*^
Normal	133 (65.5%)	48 (59.3%)	85 (69.7%)	
Overweight	39 (19.2%)	7 (8.6%)	32 (26.2%)	
**Height (cm)**				
Available data	83 (27.2%)	72 (86.7%)	11 (5.0%)	
Overall median (IQR)	161 (155, 168)	160 (156, 166)	168 (155, 175)	0.058
Male; Median (IQR)	166 (161, 170)	165 (161, 170)	173 (168, 177)	0.009^*^
Female; Median (IQR)	155 (150, 158)	155 (151, 159)	150 (149, 155)	0.190
**Duration of ART (years)**				
Overall median (IQR)	4.1 (2.4, 6.9)	15.4 (13.0, 16.6)	3.3 (1.9, 4.5)	<0.001^*^
**Current ART regimens**				
NNRTI‐based	179 (58.7%)	42 (50.6%)	137 (61.7%)	<0.001^*^
PI‐based	60 (19.7%)	31 (37.4%)	29 (13.1%)	
INSTI‐based	64 (21.0%)	9 (10.8%)	55 (24.8%)	
Others	2 (0.6%)	1 (1.20%)	1 (0.4%)	
**Remaining in initial ART regimens**				
Yes	142 (46.6%)	17 (20.5%)	125 (56.3%)	<0.001^*^
**Current CD4 cell count**				
Overall median (IQR)	538 (395, 745)	568 (353, 777)	531 (403, 717)	0.879
**Viral suppression (copies/ml)**				
Available data	294 (96.4%)	80 (96.4%)	214 (96.4%)	
Less than 200	254 (86.4%)	73 (91.3%)	181 (84.6%)	0.138
More than 1000	255 (86.7%)	74 (92.5%)	181 (84.6%)	0.075
**History of worst WHO Staging**				
Available data	92 (30.2%)	73 (88.0%)	19 (8.6%)	
Stage 1	2 (2.2%)	2 (2.7%)	0 (0%)	<0.001^*^
Stage 2	24 (26.1%)	24 (32.9%)	0 (0%)	
Stage 3	18 (19.5%)	17 (23.3%)	1 (5.3%)	
Stage 4	48 (52.2%)	30 (41.1%)	18 (94.7%)	
**Total cholesterol (mg/dl)**				
Available data	167 (54.8%)	55 (66.3%)	112 (50.5%)	
Overall median (IQR)	168 (145, 188)	181 (143, 198)	163 (145, 186)	0.090
Less than 200	28 (16.8%)	12 (21.8%)	16 (14.3%)	0.221
**Triglyceride (mg/dl)**				
Available data	144 (47.2%)	50 (60.2%)	94 (42.3%)	
Overall median (IQR)	95 (76, 141)	113 (84, 168)	90 (69, 114)	0.008^*^
More than 150	33 (22.9%)	19 (38.0%)	14 (14.9%)	0.002^*^
**High‐density lipoprotein (mg/dl)**				
Available data	127 (41.6%)	45 (54.2%)	82 (36.9%)	
Overall median (IQR)	46 (40, 57)	46 (39, 56)	47 (40, 57)	0.860
Low of HDL (female < 50, male < 40)	41 (32.3%)	17 (37.8%)	24 (29.3%)	0.327
**Low‐density lipoprotein (mg/dl)**				
Available data	37 (12.1%)	36 (43.4%)	1 (0.5%)	
Overall median (IQR)	101 (83, 118)	100 (81, 119)	118 (118, 118)	0.399
More than 130	7 (18.9%)	7 (19.4%)	0 (0%)	0.999
**Fasting blood sugar (mg/dl)**				
Available data	234 (76.7%)	46 (55.4%)	188 (84.7%)	
Overall median (IQR)	90 (85, 96)	86 (79, 94)	92 (86, 97)	<0.001^*^
FBS ≥ 100	35 (15.0%)	5 (10.9%)	30 (16.0%)	0.386
FBS ≥ 126	1 (0.4%)	0 (0%)	1(0.53%)	0.999

Abbreviations: ART, antiretroviral therapy; FBS, fasting blood sugar; HDL, high‐density lipoprotein; HIV, human immunodeficiency virus; INSTI, integrase strand transfer inhibitor; IQR, interquartile range; kg/m^2^, kilogram per square metre; mg/dl, milligrams per decilitre; ml, millimetre; mmHg, millimetre of mercury; NNRTI, non‐nucleoside reverse transcriptase inhibitor; PI, protease inhibitor; WHO, World Health Organization.

^a^ Other countries include Cambodia, China, Hong Kong SAR, Indonesia, Japan, Philippines, Singapore, South Korea, Taiwan, Vietnam, and Australia and New Zealand.

* *p* value <0.05.

After adjusting for potential confounding factors, viral suppression at age 25 was more likely among individuals living in Thailand (adjusted odds ratio [AOR] 6.05, 95% confidence interval [CI] 1.95−18.80), those with higher CD4 counts (AOR 1.00, 95% CI 1.00−1.01), those using INSTI‐based regimens (AOR 5.20, 95% CI 1.62−16.65) and those using PI‐based regimens (AOR 2.62, 95% CI 1.01−6.79). The route of HIV acquisition was not associated with viral suppression (AOR 1.22, 95% CI 0.38−3.93, *p =* 0.741) (Table 3).

**Table 3:** Factors associated with viral suppression (≤200 copies/ml) among young adults with perinatally acquired HIV at 25 years of age

Number of subjects; *n* (%)	40 (13.7)	252 (86.3)	292 (100)		292 (100)	
Characteristics	No viral suppression	Viral suppression	Odds ratio (95% CI)	*p* value	Adjusted OR (95% CI)	*p* value
**Sex**; *n* (%)						
Female	15 (37.5)	58 (23.0)	*Reference*		*Reference*	
Male	25 (62.5)	194 (77.0)	2.01 (0.99, 4.06)	0.053	1.92 (0.83, 4.42)	0.126
**Country**; *n* (%)						
Others	33 (82.5)	135 (53.6)	*Reference*		*Reference*	
Thailand	7 (17.5)	117 (46.4)	4.09 (1.74, 9.60)	0.001^*^	6.05 (1.95, 18.80)	0.002^*^
**Type of HIV acquisition**; *n* (%)						
Perinatally acquired	7 (17.5)	72 (28.6)	*Reference*		*Reference*	
Sexually acquired	33 (82.5)	180 (71.4)	0.53 (0.22, 1.26)	0.149	1.22 (0.38, 3.93)	0.741
**Current ART regimens**; *n* (%)						
NNRTI‐based	30 (75.0)	139 (55.2)	*Reference*		*Reference*	
INSTI‐based	4 (10.0)	60 (23.8)	3.24 (1.09, 9.61)	0.034^*^	5.20 (1.62, 16.65)	0.006^*^
PI‐based	6 (15.0)	53 (21.0)	1.91 (0.75, 4.85)	0.175	2.62 (1.01, 6.79)	0.047^*^
**CD4 count;** Median (IQR)	482 (232, 693)	558 (421, 756)	1.00 (1.00, 1.02)	0.018^*^	1.00 (1.00, 1.01)	0.010^*^

Abbreviations: ART, antiretroviral therapy; CI, confidence interval; HIV, human immunodeficiency virus; INSTI, integrase strand transfer inhibitor; IQR, interquartile range; NNRTI, non‐nucleoside reverse transcriptase inhibitor; OR, odds ratio; PI, protease inhibitor.

* *p* value <0.05.

## 4 DISCUSSION

Our results demonstrate variations in clinical characteristics between young adults with PHIV compared to those with sexually acquired HIV during late adolescence (18 years of age) and young adult life (25 years of age). The substantially higher proportion of males among those with sexually acquired HIV is consistent with epidemiologic trends showing a predominance of men who have sex with men among those with new HIV acquisitions in the Asia‐Pacific region [12]. Although we found individuals with PHIV experienced more advanced clinical stages, lower height and longer durations of ART than those with sexually acquired HIV, both groups had similar proportions with viral suppression.

Several studies have demonstrated substantial growth deficits among children and adolescents living with HIV compared to those without HIV [13, 14, 15]. In the present study, we noted that height deficits among those with PHIV can persist through to adulthood. Surprisingly, the deficit did not persist among females. Although early ART may improve future growth outcomes, we may need more time to see its impact in adults with PHIV.

In terms of treatment outcomes, we did not observe differences in the proportions with viral suppression at both ages. Interestingly, significantly higher levels of CD4 cell counts were found among those with PHIV at age 18. These findings may be related to a survivor effect among those with PHIV who had better adherence or the potency of available ART regimen. In our study, although the proportions of participants who had HIV viral suppression in both groups ranged from a median of 85–93%, this remains lower than the global UNAIDS target. Ensuring that adolescents and young adults with HIV have access to the most potential ART regimens remains a priority. Long‐acting injectable ART or other non‐user‐dependent dosing techniques could be options to improve virologic outcomes.

The prevalences of elevated total cholesterol and hypertriglyceridemia among those with PHIV in our study were 20% and 29% at age 18 and 22% and 38% at age 25, respectively, which were higher than those with sexually acquired HIV, but comparable to other cohorts [4]. These could be explained by longer exposure to older‐generation antiretroviral drugs associated with dyslipidaemia, such as ritonavir‐boosted protease inhibitors or stavudine. Nonetheless, the difference in hypercholesterolemia was no longer observed at age 25, which may be attributed to treatment with newer antiretrovirals. It is known that HIV itself and traditional risk factors (e.g. diabetes, hypertension, dyslipidaemia, smoking) result in a higher risk of cardiovascular disease in adults [16, 17, 18]. A recent study in South African adolescents with PHIV also showed an increased risk of atherosclerosis, predicted using the Pathobiological Determinants of Atherosclerosis in Youth (PDAY) scoring system [19]. This highlights the need to monitor for and appropriately manage metabolic complications in this population.

In the present study, living in Thailand, the use of INSTI and PI‐based regimens and CD4 cell count were associated with viral suppression at age 25. The results of our analysis were in line with other studies showing the higher potency of INSTIs as compared to NNRTI‐based regimens [20, 21], and the higher genetic barrier to resistance of PIs [22]. The association with living in Thailand may be related to the country's longer history of implementing ART through a national universal healthcare system. This highlights the vital need to improve the availability and accessibility of ART in general and of novel antiretroviral medicines to achieve better health outcomes for individuals living with HIV across all ages.

Our study was limited by the study design and data availability, leading to our inability to include other factors potentially associated with HIV viral suppression in the analysis (e.g. adherence, socio‐economic factors, mental health) [23, 24]. The number of young adults with sexually acquired HIV at age 18 was very small, resulting in the focus on those at age 25 for the multivariable analysis. The limited sample size of those with PHIV at age 25 was also reflected in the large confidence intervals. Additionally, with over half of those at age 18 being from Thailand, the findings may have limited generalizability in other contexts.

## 5 CONCLUSIONS

Clinical differences between those with PHIV and sexually acquired HIV emerged at the transition to young adulthood in our regional cohorts. Managing chronic complications of ART and lifelong HIV, including hypercholesterolemia and stunting, are emerging as priorities for adolescents with PHIV. With viral suppression rates below global targets, optimizing treatment and adherence support is essential for all young people living with HIV.

## AUTHORS’ CONTRIBUTIONS

PP and KC conceptualized and designed the study. AM cleaned and analysed data. PP and KC drafted the original manuscript. All authors contributed to the interpretation of the findings, provided critical review and revisions, and approved the final version.

## COMPETING INTERESTS

AHS receives grants to her institution from ViiV Healthcare. All other authors have no competing interests to declare.

## FUNDING

The TREAT Asia HIV Observational Database and the TREAT Asia Pediatric HIV Observational Database are initiatives of TREAT Asia, a programme of amfAR, The Foundation for AIDS Research, with support from the U.S. National Institutes of Health's National Institute of Allergy and Infectious Diseases, the *Eunice Kennedy Shriver* National Institute of Child Health and Human Development, the National Cancer Institute, the National Institute of Mental Health, the National Institute on Drug Abuse, the National Heart, Lung, and Blood Institute, the National Institute on Alcohol Abuse and Alcoholism, the National Institute of Diabetes and Digestive and Kidney Diseases, and the Fogarty International Center, as part of the International Epidemiology Databases to Evaluate AIDS (IeDEA; U01AI069907). The Kirby Institute is funded by the Australian Government Department of Health and Ageing, and is affiliated with the Faculty of Medicine, UNSW Sydney.

## DISCLAIMER

The content of this publication is solely the responsibility of the authors and does not necessarily represent the official views of any of the governments or institutions mentioned above.

## Data Availability

The data that supported this study are not openly available due to their containing sensitive information (e.g. sex, date of birth, date of death) that could compromise the privacy of research participants. Data are formally owned by the contributing clinical sites. De‐identified aggregated data are, however, available with permission of the study Steering Committee and request to access will be subject to HREC review of all the participating sites prior to sharing. Data requests may be sent to study project manager, Tulathip Suwanlerk (tulathip.suwanlerk@treatasia.org) at, TREAT Asia, Bangkok, Thailand, or the study data manager, Azar Kariminia (Akariminia@kirby.unsw.edu.au), at the Kirby Institute, UNSW, Sydney, Australia, which serves as the study's regional data centre.
